# Supplementary material for: The STAT3/Slug Axis Enhances Radiation-Induced Tumor Invasion and Cancer Stem-like Properties in Radioresistant Glioblastoma
Source: Cancers (Basel). 2018 Dec 13;10(12):512. doi: 10.3390/cancers10120512 (PMC6315497; doi:10.3390/cancers10120512)
Supplement: Supplementary file 1 [file cancers-10-00512-s001.pdf]

Supplementary Materials: The STAT3/Slug Axis Enhances Radiation-Induced Tumor Invasion and Cancer Stem-like Properties in Radioresistant Glioblastoma

Jang-Chun Lin, Jo-Ting Tsai, Tsu-Yi Chao, Hsin-I Ma and Wei-Hsiu Liu

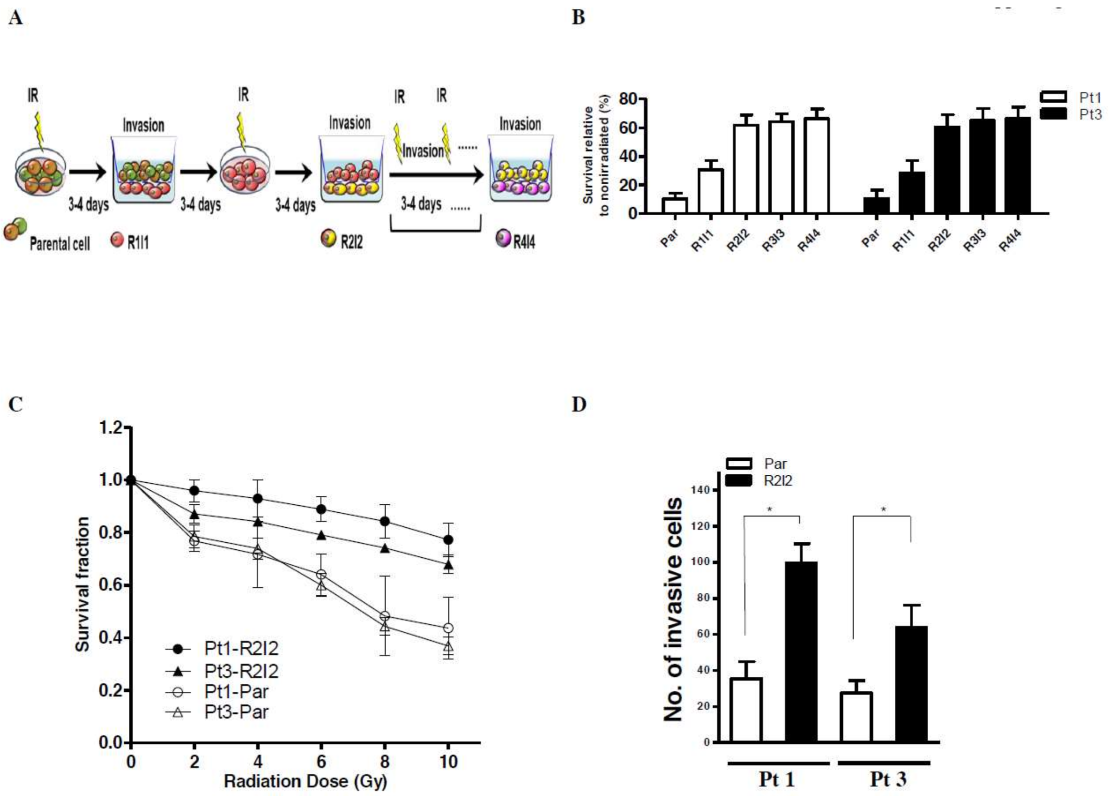

Figure S1. Radioresistant GBM cells display a more invasive phenotype. (**A**) Representative radio-resistant picture of GBM. The primary GBM cell lines received ionizing radiations (IR) and then irradiated cells also received transwell invasion assay. Then the irradiated/ invasinve cells were generated for several cell lines, termed Par, R1I1, and R2. R4I4. (**B**) Cell viability of survical relative rate to nonirradiaated cell in the cell lines, Par, R1I1, and R2. R4I4. (**C**) GBM-Par and GBM-R2I2 cells in two individual patients were subjected to clongenic assays to assess the glioblastoma cells phenotype. Scale bars: 50 μm. * $p < 0.01$ by Student's t -test. (**D**) GBM-Par and GBM-R2I2 cells in two individual patients were subjected to invasion assays to assess the glioblastoma cells phenotype. * $p < 0.01$ by Student's t -test.

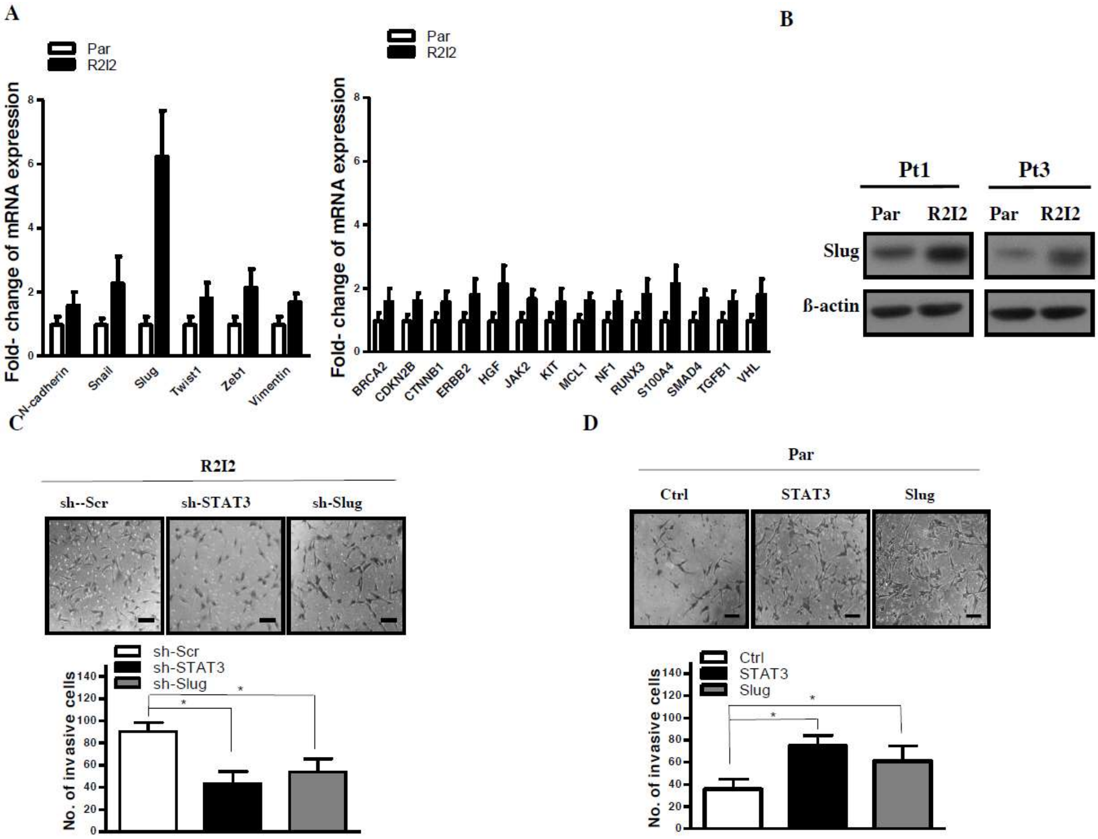

Figure S2. STAT3 activates cell motility and tumor invasion through Slug. (**A**)Left: A qPCR analysis of EMT-related genes N-cadherin, Snail, Slug, Twist1, Zeb1 and Vimentin. Right: A qPCR analysis ofRT2Profiler PCR Arraygenes. (**B**) Western blot of the target gene Slug. (**C**) Transwell invasion assay in GBM-R2I2 cells transfected with sh-STAT3 or sh-Slug versus scrambled shRNA control vector (sh-Scr). Scale bars, 50 μm. * $p < 0.01$ by Student's -test. (**D**) Transwell invasion assay in GBM-Par cells transfected with ectopic STAT3 or Slug versus the vector control (Ctrl). Scale bars: 50 μm.* $p < 0.01$by Student's *t*–test. The data shown are the mean SD of three independent experiments.

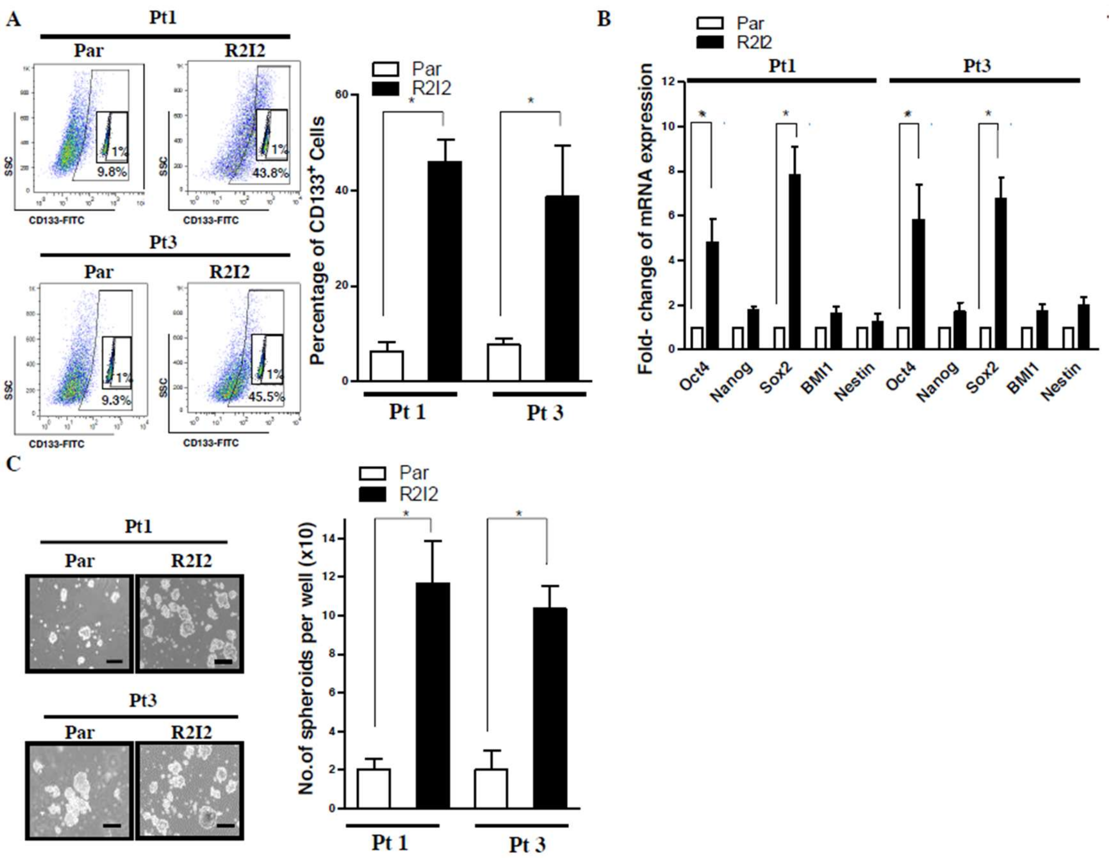

Figure S3. The STAT3/Slug axis acquires the stemness and tumor-initiating capacities in GBM-R2I2 cells. (**A**) The presence of CD133+ positive cells of GBM-Par cells compared with that of GBM-R2I2 cells by flow cytometry. * $p < 0.05$ by Student's t -test. The data shown are the mean ± SD of three independent experiments. (**B**) A qPCR analysis of Oct4, Nanog, Sox2, BMI-1, and Nestin in GBM-Par cells compared with GBM-R2I2 cells. * $p < 0.01$ by Student's *t* -test. (**C**) In sphere-forming assay, GBM-R2I2 cells acquire higher sphere-forming numbers than GBM-Par cells. Scale bars: 50μm. * $p < 0.01$ by Student's *t* -test. *$p < 0.01$ by Student's *t*-test. The data shown are the mean ± SD of three independent experiments.

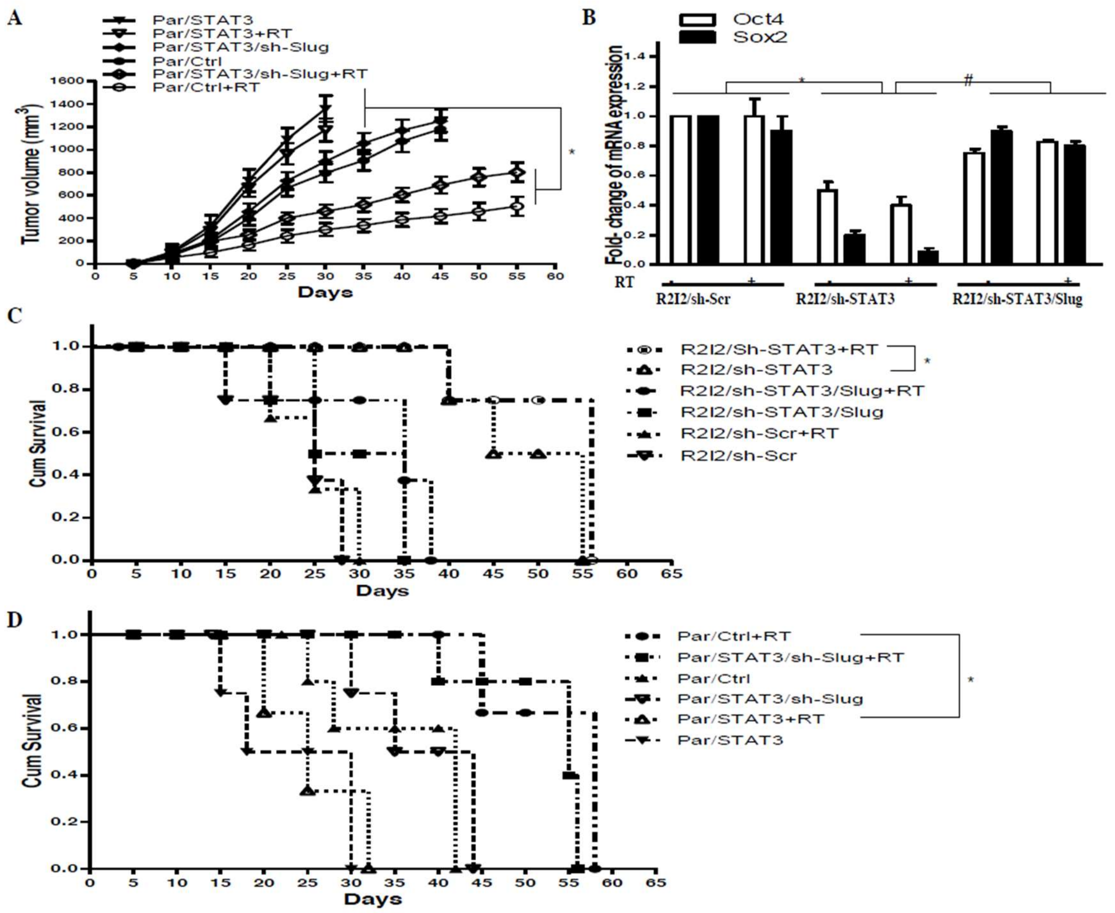

Figure S4. STAT3/ Slug axis silencing increases the synergistic effects with radiosensitivity and prolongs the survival of GBM-R2I2 in vivo. GBM-Par were intracranially transplanted into NOD-SCID mice, and six mice in each group (*n* = 6 in each group; total 36 mice). (**A**) Tumor volumes in GBM-Par r transplanted mice treated with vector control (Ctrl) combined with IR (5Gy) treatment were significantly smaller than those receiving different protocol. * $p < 0.01$ by Student's *t*-test. (**B**) A qPCR analysis of Oct4, and Sox2 in R2I2/sh-Scr, R2I2/sh-STAT3, and R2I2/sh-STAT3/Slug cells with or without IR in transplanted mice. * $p < 0.01$ by Student's *t*-test. (**C**) Kaplan-Meier survival analysis further described mean survival rate for animals injected with GBM-R2I2 cells treated with indicated treatments. Mice with GBM-R2I2 cells treated with sh-STAT3 and IR had a significantly prolonged survival rate compared with untreated GBM-R2I2 mice. * $p < 0.01$ by log rank test. The data shown are the mean ± SD of three independent experiments. (**D**)Kaplan-Meier survival analysis further revealed that the mean survival rate for animals injected with GBM-Par treated with indicated treatments. * $p < 0.01$ by Student's *t*-test. The data shown are the mean ± SD of three independent experiments.

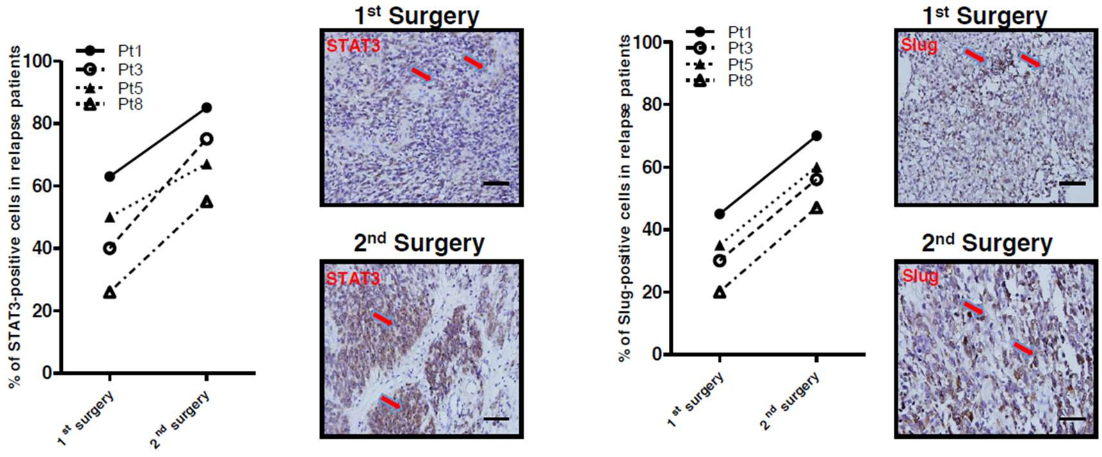

Figure S5. The percentage of STAT3-and Slug-positive GBM cells (1st surgery, 9 patients) was dramatically elevated in the tumor-relapse samples (2nd surgery, 4 patients).

Table S1. Primers for Slug promoter constructions, ChIP and Q-ChIP.

Primers for Slug promoter constructions, specific PCR, ChIP		
**Slug**	**Slug** Full F	5´-AGTCTTGACATCACCACTGT-3´
	**Slug** Full R	5´-GGCTGGGAGGGTTTTTTTT-3´
	**Slug**-D1 F	5´-AATTTGTTCTTTCCTTATTCGATAGGGATA-3´
	**Slug**-D2 F	5´-TCTTCCCGCTTCCCCCTTCCGCCAAGAGGT-3´
	**Slug**-D3 F	5´-CTCTCAGCTGTGATTGGATCGAGAGGAAAA-3´
	Mut **Slug** F	5´-CCCCCTTCCTTTTCAAGGGCCAAGAGGTAA-3´
	Mut **Slug** R	5´-TTACCTCTTGGCCCTTGAAAAGGAAGGGGG-3´
ChIP and Q-ChIP for **Slug**	-38~-27 F	5´-CAAACCACTGTACAAAGAATTGTTTGTT-3´
	-38~-27 R	5´-TACAGTGGTTTGGTACTAATCATG-3´
	-472~-463 F	5´-TTTTTCAAAAGCCAAGAGGTAATTATT-3´
	-472~-463 R	5´-TTTTGAAAAAGGAAGGGGGAAGCGG-3´
	-1195~-1185 F	5´-TTTTAGCAAAAGATAGGGATAAAAGTC-3´
	-1195~-1185 R	5´-TTTTGCTAAAAGAATAAGGAAAGAA-3´
	N. C. F	5´-ACCTGTTAGAAACAAGAGTA-3´
	N. C. R	5´-TCTAACAGGTGCTGGAGGAA-3´

ChIP: chromatin immunoprecipitation. N.C: Non-specific control region.

Table S2. The sequences of the primers for quantitative RT-PCR.

**Gene (Accession No.)**	**Primer Sequence (5´ to 3´)**	**Product size (bp)**	**Tm (°C)**
STAT3 (NM_003150)	F: AGCAGCACCTTCAGGATGTC<br>R: GCATCTTCTGCCTGGTCACT	168	60
Slug (NM_003068)	F: GTGATTATTTCCCCGTATCTCTAT<br>R: CAATGGCATGGGGGTCTGAAAG	292	55
Snail (NM_005985)	F: CGAGCTGCAGGACTCTAAT<br>R: CCACTGTCCTCATCTGACA	231	55
BRCA1 (NM_007294)	F: TGTGAGGCACCTGTGGTGA<br>R: CAGCTCCTGGCACTGGTAGAG	69	55
Rac1 (NM_006908)	F: CACGATCGAGAAACTGAAGGA<br>R: AGCAGGCATTTTCTCTTCCTC	201	58
Rho (NM_000539)	F: GAAGCCACCTGCTCTTTTGC<br>R: CAAGGAAGGTAGGCCCAGTG	174	55

N-cadherin (NM_001792)	F: CCACGCCGAGCCCCAGTATC<br>R: CCCCCAGTCGTTCAGGTAATCA	232	61
Twist1 (NM_000474)	F: GGGAGTCCGCAGTCTTACGA<br>R: AGACCGAGAAGGCGTAGCTG	277	61
Zeb1 (NM_030751)	F: ACTGCTGGGAGGATGACAGA<br>R: ATCCTGCTTCATCTGCCTGA	72	55
Vimentin (NM_003380)	F: GCAATCTTTCAGACAGGATGTTGAC<br>R: GATTTCCTCTTCGTGGAGTTTCTTC	118	59
Oct-4 (NM_002701)	F: TGTGGACCTCAGGTTGGACT<br>R: CTTCTGCAGGGCTTTCATGT	207	58
Nanog (NM_024865)	F: TCTTCCTACCACCAGGGATGC<br>R: CACTGGCAGGAGAATTTGGC	250	59
Sox2 (NM_003106)	F: CGAGTGGAAACTTTTGTCGGA<br>R: TGTGCAGCGCTCGCAG	74	58
Nestin (NM_006617)	F: AGGAGGAGTTGGGTTCTG<br>R: GGAGTGGAGTCTGGAAGG	112	55
Bmi1 (NM_ 005180)	F:AAATGCTGGAGAACTGGAAAG<br>R:CTGTGGATGAGGAGACTGC	124	57
GAPDH (NM_002046)	F: CATCATCCCTGCCTCTACTG<br>R: GCCTGCTTCACCACCTTC	180	58

Bp, base pairs; Sox2, sex determining region Y-box 2; GAPDH, glyceraldehyde 3-phosphate dehydrogenase.

Table S3. List of proteins tested by antibodies.

**Protein**	**Assay**	**Antibody**	**Origin**	**Dilution**	**Incubation period**
STAT3	WB<br>IF<br>IHC	mmab	#9139, Cell Signaling, Inc	1:1000<br>1:1000<br>1:500	overnight
p-STAT3	WB	mmab	#4113, Cell Signaling, Inc	1:1000	overnight
Slug	WB	rpab	Ab38551, Abcam, Inc	1:1000	overnight
BCAR1	WB	rpab	Ab80016, Abcam, Inc	1:1000	overnight
Rac1	WB	mmab	Ab33186, Abcam, Inc	1:1000	overnight.
Rho	WB	rmab	Ab17732, Abcam, Inc	1:2000	overnight
N-cadherin	WB<br>IF	rpab	Ab18203, Abcam, Inc	1:1000<br>1:200	overnight
E-cadherin	WB<br>IF	mmab	Ab76055, Abcam, Inc	1:1000<br>1:200	overnight
Snail	WB<br>IHC	rpab	Ab180714, Abcam, Inc	1:1000<br>1:200	overnight
Twist1	WB	rpab	#4119, Cell Signaling, Inc	1:1000	overnight
Zeb1	WB	mmab	Ab180905, Abcam, Inc	1:2000	overnight
Vimentin	WB	rpab	#4745, Cell Signaling, Inc	1:1000	overnight
Fibronectin	IF	rpab	Ab2413, Abcam, Inc	1:200	2hrs
ß-actin	WB	mmab	Ab3280, Abcam, Inc	1:10000	

Abbreviations: WB, Western blot; mmab, mouse monoclonal antibody; rmab, rabbit monoclonal antibody; rpab, rabbit polyclonal antibody ;IF, immunofluorescence; IHC, Immunohistochemistry.

Table S4. Primers for 6×RE STAT3 binding sites reporter construction.

Primers for 6xRE STAT3 binding sites reporter construction		
6xRE STAT3	Forward synthesized 5′-phosphorylated	5′-pTTACTCTGAAAATTACTCTGAAAATTACTCTGAAAAT TACTCTGAAAA TTACTCTGAAAATTACTCTGAAAA-3′
	Reverse synthesized 5′-phosphorylated	5′-pTTTTCAGAGTAATTTTCAGAGTAATTTTCAGAGTAA TTTTCAGAGTAATTTTCAGAGTAATTTTCAGAGTAA-3′
Mutated 6xRE STAT3	Forward synthesized 5′-phosphorylated	5′-pTTACTCTGGGAATTACTCTGGGAATTACTCTGGGAAT TACTCTGGGAA TTACTCTGGGAATTACTCTGGGAA-3′
	Reverse synthesized 5′-phosphorylated	5′-pTTCCCAGAGTAATTCCCAGAGTAATTCCCAGAGTAAT TCCCAGAGTAATTCCCAGAGTAATTCCCAGAGTAA-3′

Table S5. STAT3/Slug axis regulated the tumor-initiating activity of GBM in vivo.

Pt. No.	Injected Cells Numbers	R2I2/sh-Scr	R2I2/sh-STAT3	R2I2/sh-STAT3 + Slug	Par/Ctrl	Par/STAT3	Par/STAT3 + sh-Slug
Pt. 1	50,000	3/3	3/3	3/3	3/3	3/3	3/3
	10,000	3/3	2/3	3/3	2/3	3/3	2/3
	1,000	3/3	1/3	3/3	0/3	3/3	2/3
	500	2/3	0/3	1/3	0/3	0/3	0/3
	100	2/3	0/3	1/3	0/3	0/3	0/3
	50	0/3	0/3	0/3	0/3	0/3	0/3
Pt. 2	50,000	3/3	3/3	3/3	1/3	3/3	3/3
	10,000	3/3	1/3	3/3	2/3	2/3	1/3
	1,000	2/3	1/3	1/3	0/3	1/3	1/3
	500	0/3	0/3	1/3	0/3	0/3	0/3
	100	0/3	0/3	0/3	0/3	0/3	0/3
	50	0/3	0/3	0/3	0/3	0/3	0/3

GBM tumor- R2I2/sh-Scr, R2I2/sh-STAT3, R2I2/sh-STAT3+Slug, Par/Ctrl,. Par/STAT3 and Par/STAT3+sh-Slug transfected cells were transplanted into the brain striatum of mice with different number of cells as indicated (N = 3). Each GBM tumor cell type wasinjected into 18 mices.After 8 weeks follow. After 8 weeks follow-up, the presence of tumor nodules in each mouse was determined and listed in the table.

Table S6. GBM patients' description and characteristics.

Patient No.	Age/Sex	Treatment	Survival time
1	57/M	1st Surgery + CCRT + 2nd surgery	1.0 yr
2	83/M	1st Surgery+ CCRT	0.8 yr
3	69/F	1st Surgery + CCRT + 2nd surgery	2.3 yr
4	75/F	1st Surgery + CCRT	1.8 yr
5	45 /M	1st Surgery + CCRT + 2nd surgery	3.7 yr
6	56/M	1st Surgery + CCRT	3.2 yr
7	63/M	1st Surgery + CCRT + 2nd surgery	1.4 yr
8	48/M	1st Surgery + CCRT + 2nd surgery	2.7 yr
9	71/F	1st Surgery + CCRT	1.5 yr

The second surgery for tumor relapses.

© 2018 by the authors. Licensee MDPI, Basel, Switzerland. This article is an open access article distributed under the terms and conditions of the Creative Commons Attribution (CC BY) license (http://creativecommons.org/licenses/by/4.0/).
